# A comparison of physician emigration from Africa to the United States of America between 2005 and 2015

**DOI:** 10.1186/s12960-017-0217-0

**Published:** 2017-06-26

**Authors:** Robbert J. Duvivier, Vanessa C. Burch, John R. Boulet

**Affiliations:** 1 Foundation for Advancement of International Medical Education and Research (FAIMER), Philadelphia, United States of America; 2 Medical Education Unit, School of Medicine and Public Health, University of Newcastle, University Drive, Callaghan, NSW 2308 Australia; 3 Department of Medicine, Faculty of Health Sciences, University of Cape Town, Cape Town, South Africa

**Keywords:** International medical graduates, Medical education, Medical schools, Africa, Migration, Brain drain

## Abstract

**Background:**

Migration of health professionals has been a cause for global concern, in particular migration from African countries with a high disease burden and already fragile health systems. An estimated one fifth of African-born physicians are working in high-income countries. Lack of good data makes it difficult to determine what constitutes “African” physicians, as most studies do not distinguish between their country of citizenship and country of training. Thus, the real extent of migration from African countries to the United States (US) remains unclear. This paper quantifies where African migrant physicians come from, where they were educated, and how these trends have changed over time.

**Methods:**

We combined data from the Educational Commission for Foreign Medical Graduates with the 2005 and 2015 American Medical Association Physician Masterfiles. Using a repeated cross-sectional study design, we reviewed the available data, including medical school attended, country of medical school, and citizenship when entering medical school.

**Results:**

The outflow of African-educated physicians to the US has increased over the past 10 years, from 10 684 in 2005 to 13 584 in 2015 (27.1% increase). This represents 5.9% of all international medical graduates in the US workforce in 2015. The number of African-educated physicians who graduated from medical schools in sub-Saharan countries was 2014 in 2005 and 8150 in 2015 (304.6% increase). We found four distinct categorizations of African-trained physicians migrating to the US: (1) citizens from an African country who attended medical school in their own country (86.2%, *n =* 11,697); (2) citizens from an African country who attended medical school in another African country (2.3%, *n =* 317); (3) US citizens who attended medical school in an African country (4.0%, *n =* 537); (4) citizens from a country outside Africa, and other than the United States, who attended medical school in an African country (7.5%, *n =* 1013). Overall, six schools in Africa provided half of all African-educated physicians.

**Conclusions:**

The number of African-educated physicians in the US has increased over the past 10 years. We have distinguished four migration patterns, based on citizenship and country of medical school. The majority of African graduates come to the US from relatively few countries, and from a limited number of medical schools. A proportion are not citizens of the country where they attended medical school, highlighting the internationalization of medical education.

## Background

The large-scale emigration of highly skilled and well-educated workers has been likened to a “brain drain” [1]. Migration of health professionals in particular has been a cause for global concern [2], in particular migration from African countries with a high disease burden and already fragile health systems. Among others, the World Health Organization [3] and the US Institute of Medicine [4] have noted the negative consequences of midwives, nurses, and physicians leaving Africa. An estimated one fifth of African-born physicians are working in high-income countries^1^. The top four recipient countries employing doctors trained elsewhere are Australia, Canada, the United Kingdom (UK), and the United States (US) [5]. These four countries derive around one quarter of their workforce from international medical graduates (IMGs), the majority emigrating from lower-income countries [6]. Of these four recipient nations, the US employs three quarters of all IMGs worldwide. The US has relied on IMGs to supplement its physician workforce since the 1950s [6], with the number of internationally trained doctors tripling from 70 646 in 1973 [7] to 210 000 in 2003 [8]. The estimated number of African IMGs in the US in 2004 was 5334 (2002 data [8], from a subset of sub-Saharan countries); in 2013, there were 17 376 physicians either born or trained in Africa in the US physician workforce (2011 data [9]).

In light of these figures, public and academic attention has centered on the ethics of employing doctors who have been trained in lower-income countries [10, 11]. However, despite the plethora of literature on health worker migration, there is a remarkable paucity and incompleteness of data [12, 13]. Moreover, most of the literature on African IMGs has tended to focus on a particular sub-region, namely sub-Saharan Africa [7, 8, 14, 15]. Such previous studies that address migration have treated all graduates from an African medical school as African IMGs, with no distinction between an individual’s country of citizenship and country of training. This makes it difficult to determine the real scope of migration from African countries to the US, or other nations, and thus develop meaningful national or international workforce policies [16, 17].

The purpose of this paper is to provide a profile of African-trained physicians working in the US, reviewing both historical patterns and recent emigration trends. Our methodology differs from previous studies [8, 9] in that we combined data sources to obtain information at both the school level and the individual level. We specifically investigated the citizenship status of African-trained physicians who sought educational and practice opportunities in the US.

## Methods

We obtained information about all African-trained physicians working in the US from the 2005 and 2015 American Medical Association (AMA) Physician Masterfiles [18]. The AMA Masterfile contains information on physicians practicing in the US, including type of practice and major professional activity. We used data from physicians who were active, including those who are employed in non-clinical positions such as research, teaching, or administration. To be listed in the AMA Masterfile, an IMG must have started a residency program in the US. Using a unique identifier, we combined individual AMA records with demographic information available from the Educational Commission for Foreign Medical Graduates (ECFMG). The ECFMG certifies all IMGs who wish to obtain residency positions in the US; for IMGs, ECFMG certification and residency training is required to obtain an unrestricted license to practice medicine. As part of the certification process, ECFMG collects demographic information, including citizenship at entry to medical school. For the purpose of this paper, we defined international medical graduates (IMGs) as individuals who attended medical schools located outside the US or Canada. Physicians who were educated in Africa, regardless of citizenship at entry to medical school, are referred to as African-educated IMGs. Those who were educated in a medical school in sub-Saharan Africa (SSA), regardless of citizenship, were designated as SSA IMGs. The designation SSA is used to indicate all of Africa except northern Africa, with the Sudan included in sub-Saharan Africa, as specified in the statistics of UN institutions.^1^ Using a cross-sectional approach, we reviewed the available data, including medical school attended, country of medical school, and citizenship at entry to medical school. Data analysis was done with SAS v9 [19].

## Results

**Fig. 1:**
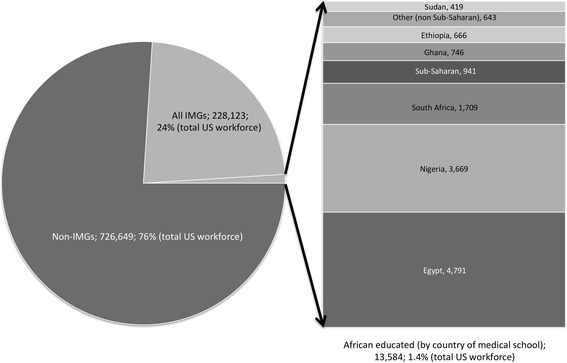
IMGs in the US, 2015 data

Between 2005 and 2015, the active physician pool in the US increased from 836 190 to 954 772, or 14.2%. Figure 1 shows that of all the physicians active in the US in 2015, including those in residency programs, administration, and research and teaching positions, 228 123, or 24%, are IMGs. Altogether, 13 584 of these IMGs (5.9% of all IMGs in the US) had been educated at an African medical school. In the same time period, the number of African-educated IMGs grew from 10 684 to 13 584, a 27.1% increase. This represents a net increase of 2900 African-educated physicians since 2005, or about one African-educated doctor migrating to the US per day over the last decade.

In 2015, half (50.2%) of all IMGs in the US workforce were educated in seven countries; India, Philippines, Mexico, Pakistan, Dominican Republic, Grenada, Egypt. India alone accounted for 22.1% of all IMGs in the US in 2015. Egypt, a Northern African (non-SSA) country, is the only African nation in the top 10 source countries, and accounted for 4223 IMGs in 2005 (2.3% of US workforce) and 4791 in 2015 (2.1% of workforce).

Table 1 shows the country of medical school for all African-educated physicians active in the US workforce in 2005 and 2015. Four countries (Egypt, Nigeria, South Africa, and Ghana) account for the majority of African emigration: in 2015, 86.0% of all African-educated IMGs in the US attended medical school at institutions in these nations. In 2015, the total number of IMGs who were educated in medical schools in SSA countries was 8150; this was 2014 more doctors than in 2005. In 2015, those IMGs who graduated from SSA medical schools represented 60.0% of the total African-educated contingent, with Nigeria, South Africa and Ghana accounting for three quarters (*n =* 6124). Of the increase in African-educated physicians practicing in the US since 2005, 1156 were educated in Nigeria, South Africa, and Ghana—leaving a net increase of 858 SSA-educated physicians from countries other than these three in the US between 2005 and 2015.

**Table 1:** African-educated IMGs in the US physician workforce by country of medical school

	2005		2015			
	Number	% of African-educated physicians	Number	% of African-educated physicians	Change	% Change
ALGERIA	86	0.80	107	0.79	21	+24.42
**ANGOLA**			1	0.01	1	
**BENIN**	1	0.01	4	0.03	3	+300.00
**BURUNDI**	2	0.02	3	0.02	1	+50.00
**CAMEROON**	34	0.32	72	0.53	38	+111.76
**CONGO, DEM. REP. OF THE**	7	0.07	12	0.09	5	+71.43
**COTE D'IVOIRE**	2	0.02	8	0.06	6	+300.00
EGYPT	4 223	39.53	4 791	35.27	568	+13.45
**ETHIOPIA**	355	3.32	666	4.90	311	+87.61
**GABON**			1	0.01	1	
**GHANA**	551	5.16	746	5.49	195	+35.39
**GUINEA**	2	0.02	2	0.01	0	0
**KENYA**	133	1.24	188	1.38	55	+41.35
LIBERIA	51	0.48	54	0.40	3	+5.88
LIBYA	96	0.90	331	2.44	235	+244.79
**MADAGASCAR**	3	0.03	2	0.01	−1	−33.33
**MALAWI**	3	0.03	4	0.03	1	+33.33
**MALI**	1	0.01	1	0.01	0	0
**MAURITIUS**			29	0.21	29	
MOROCCO	64	0.60	96	0.71	32	+50.00
**NIGER**	2	0.02	4	0.03	2	+100.00
**NIGERIA**	2 575	24.10	3 669	27.01	1 094	+42.49
**RWANDA**	3	0.03	5	0.04	2	+66.67
**SENEGAL**	24	0.22	163	1.20	139	+579.17
SEYCHELLES			16	0.12	16	
**SIERRA LEONE**	4	0.04	8	0.06	4	+100.00
**SOMALIA**	11	0.10	14	0.10	3	+27.27
**SOUTH AFRICA**	1 842	17.24	1 709	12.58	−133	−7.22
**SUDAN**	215	2.01	419	3.08	204	+94.88
**TANZANIA**	18	0.17	34	0.25	+16	+88.89
**TOGO**			3	0.02	+3	
TUNISIA	28	0.26	39	0.29	+11	+39.29
**UGANDA**	177	1.66	200	1.47	+23	+12.99
**ZAMBIA**	72	0.67	74	0.54	+2	+2.78
**ZIMBABWE**	99	0.93	109	0.80	+10	+10.10
Total sub-Saharan^a^	6 136	57.43	8 150	60.00	2 014	+32.82
Total African (other than sub-Saharan)	4548	42.57	5 434	40.00	+886	+19.48
TOTAL	10,684		13,584		2 900	+27.14

^a^Nations in bold are considered sub-Saharan Africa

Table 2 provides data on the citizenship (at entry to medical school) for African-educated physicians in the US workforce.

**Table 2:** African-educated IMGs in the US physician workforce by country of citizenship at entry to medical school

	2005		2015		
	Number	% of total	Number	% of total	% change
EGYPT	3 655	34.27	Err:511	Err:511	Err:511
**NIGERIA** ^a^	2 203	20.66	Err:511	Err:511	Err:511
**SOUTH AFRICA**	1 633	15.31	Err:511	Err:511	Err:511
**GHANA**	547	5.13	Err:511	Err:511	Err:511
**ETHIOPIA**	355	3.33	Err:511	Err:511	Err:511
UNITED STATES OF AMERICA	313	2.94	Err:511	Err:511	Err:511
JORDAN	220	2.06	Err:511	Err:511	Err:511
**SUDAN**	218	2.04	Err:511	Err:511	Err:511
UNITED KINGDOM	180	1.69	Err:511	Err:511	Err:511
**KENYA**	151	1.42	Err:511	Err:511	Err:511
INDIA	140	1.31	Err:511	Err:511	Err:511
**ZIMBABWE**	113	1.06	Err:511	Err:511	Err:511
**UGANDA**	90	0.84	Err:511	Err:511	Err:511
LIBYA	78	0.73	Err:511	Err:511	Err:511
ALGERIA	77	0.72	Err:511	Err:511	Err:511
PAKISTAN	60	0.56	Err:511	Err:511	Err:511
MOROCCO	59	0.55	Err:511	Err:511	Err:511
**CAMEROON**	52	0.49	Err:511	Err:511	Err:511
SYRIA	52	0.49	Err:511	Err:511	Err:511
LEBANON	48	0.45	Err:511	Err:511	Err:511
Other sub-Saharan	173	1.62	200	1.48	+15.61
Other African (non sub-Saharan)	59	0.55	64	0.47	+8.47
Other (non-African)	188	1.76	234	1.73	+24.47
Total sub-Saharan^a^	5 535	51.90	Err:511	Err:511	Err:511
Total African (non sub-Saharan)	3 928	36.83	Err:511	34.88	Err:511
Total other (non-African)	1 201	11.26	Err:511	Err:511	Err:511

Total numbers for Table 2 differ from Table 1 because of missing citizenship information. <0.05% of the physicians did not have citizenship information coded in the ECFMG database

^a^Nations in bold are considered sub-Saharan Africa

The total number of African-educated IMGs with citizenship from a country outside Africa increased by 29.3%. Compared to 2005, in 2015 there were notably more African-educated physicians in the US with citizenship from Libya (+256.4%), Cameroon (+101.9%), Sudan (+88.1%), Ethiopia (+87.6%), and the US (+71.6%). In 2015, African-educated physicians (those who attended medical school in an African country) included citizens from 64 different non-African countries, including Middle-Eastern countries such as Jordan (*n =* 201, none of whom attended SSA schools) and Syria (*n =* 72, 3 were educated in SSA schools), Asian countries such as India (*n =* 184, 175 attended SSA schools) and Pakistan (*n =* 71, 44 attended SSA schools), the United Kingdom (*n =* 208, 199 attended SSA schools), and the US (*n =* 537, 362 attended SSA schools). In total, based on 2015 data, 1552 (11.5%) African-educated physicians held citizenship from a non-African country at entry to medical school. This leaves 11 997 African-educated IMGs who held citizenship from an African country at the time of their education; 7270 (53.7%) of whom had citizenship (at entry to medical school) of a SSA country.

For those IMGs with citizenship from an African country at entry to medical school, citizens from five countries (Egypt, Nigeria, South Africa, Ethiopia, and Ghana) account for 76.0% of all African-educated physicians in the United States (2015 data), with Egyptian (30.9%) and Nigerian (23.9%) physicians counting for half of this cohort. Of the sub-Saharan contingent, 89.7% were citizens of five countries: Nigeria (44.5%), South Africa (20.4%), Ghana (10.0%), Ethiopia (9.2%), and Sudan (5.6%). Excluding these five countries, only 747 African-educated IMGs were citizens of countries in the rest of sub-Saharan Africa.

Between 2005 and 2015, there was an over 70% increase in the number of US citizens attending African medical schools and subsequently entering the US workforce. In 2015, nearly 4% (*n =* 537) of all African-educated physicians in practice in the United States were US citizens at entry to medical school.

Table 3 shows, for 2005, the medical schools in Africa with greater than 100 graduates currently practicing medicine in the United States, while Table 4 shows this for 2015. In 2015, for half of the African-educated IMGs in the US physician workforce, their Alma Mater is one of only six schools (three in Egypt, two in Nigeria, and one in South Africa), out of a total number of 208 medical schools in Africa [20]. Based on African-educated physicians in the US in 2015, 12.5% (*n =* 1867) were non-domestic students (i.e., from a country other than that of the host university). Of these, 1550 (83.0%) were not citizens of an African country at entry to medical school; 1013 were US citizens.

**Table 3:** Medical Schools with >100 Graduates in the US physician workforce (2015 data)

	Country	Number	% of total African-educated IMGs	Number of domestic citizens^a^ (category 1)	% of school cohort	Number of non-domestic African citizens (category 2)	% of school cohort	Number of US citizens (category 3)	% of school cohort	Number of non-domestic non-African citizens (category 4)	% of school cohort
University of Cairo Faculty of Medicine	Egypt	1 680	12.39	1 466	87.26	17	1.01	52	3.10	165	9.82
Ain Shams University Faculty of Medicine	Egypt	1 439	10.61	1 262	87.70	15	1.04	48	3.34	114	7.92
University of Alexandria Faculty of Medicine	Egypt	1 063	7.84	937	88.15	17	1.60	31	2.92	78	7.34
University of the Witwatersrand Faculty of Health Sciences	South Africa	917	6.76	826	90.08	15	1.64	19	2.07	57	6.22
University of Ibadan College of Medicine	Nigeria	903	6.66	791	87.60	15	1.66	19	2.10	78	8.64
University of Nigeria Faculty of Medicine	Nigeria	697	5.14	630	90.39	5	0.72	28	4.02	34	4.88
University of Lagos College of Medicine	Nigeria	599	4.42	525	87.65	7	1.17	24	4.01	43	7.18
University of Cape Town Faculty of Health Sciences	South Africa	544	4.01	444	81.62	54	9.93	8	1.47	38	6.99
University of Ghana School of Medicine and Dentistry	Ghana	527	3.89	514	97.53	6	1.14	1	0.19	6	1.14
Addis Ababa University, College of Health Sciences, School of Medicine	Ethiopia	365	2.69	355	97.26	5	1.37	1	0.27	4	1.10
University of Benin School of Medicine	Nigeria	362	2.67	327	90.33	2	0.55	15	4.12	25	6.87
University of Khartoum Faculty of Medicine	Sudan	302	2.23	282	93.38	6	1.66	8	2.21	21	5.80
Obafemi Awolowo University College of Health Sciences	Nigeria	291	2.15	63	21.65	5	1.66	9	2.98	6	1.99
University of Tripoli Faculty of Medicine	Libya	225	1.66	184	81.78	7	3.11	18	8.00	16	7.11
Kwame Nkrumah University of Science & Technology School of Medical Sciences	Ghana	217	1.6	204	94.01	5	2.30	3	1.38	5	2.30
Assiut University Faculty of Medicine	Egypt	180	1.33	174	96.67	1	0.56	3	1.67	2	1.11
University of Ilorin College of Health Sciences	Nigeria	173	1.28	157	90.75	1	0.58	5	2.89	10	5.78
University of Nairobi School of Medicine	Kenya	161	1.19	153	95.03	4	2.48	1	0.62	3	1.86
Gondar College of Medicine and Health Sciences	Ethiopia	152	1.12	150	98.68	2	1.32		0.00		0.00
St. Christopher Iba Mar Diop College of Medicine	Senegal	151	1.11	0	0.00	3	1.99	124	82.12	24	15.89
Jimma University College of Public Health and Medical Sciences	Ethiopia	149	1.1	148	99.33		0.00	1	0.67		0.00
Makerere University School of Medicine	Uganda	142	1.05	95	66.90	37	26.06	1	0.70	9	6.34
University of Pretoria School of Medicine	South Africa	120	0.88	114	95.00	2	1.67	2	1.67	2	1.67
University of Jos Faculty of Medical Sciences	Nigeria	112	0.83	85	75.89	1	0.89	2	1.79	24	21.43
Ahmadu Bello University Faculty of Medicine	Nigeria	110	0.81	84	76.36	2	1.82	3	2.73	21	19.09
Mansoura University Faculty of Medicine	Egypt	109	0.8	101	92.66		0.00	2	1.83	6	5.50
University of Zimbabwe College of Health Sciences	Zimbabwe	109	0.8	70	64.22	9	8.26	8	7.34	22	20.18

^a^Domestic means students who hold citizenship from the same country as their medical school is located in

**Table 4:** Medical Schools with >100 Graduates in the US physician workforce (2005 data)

	Country	Number	% of total African-educated IMGs	Number of domestic citizens^a^ (category 1)	% of school cohort	Number of non-domestic African citizens (category 2)	% of school cohort	Number of US citizens (category 3)	% of school cohort	Number of non-domestic non-African citizens (category 4)	% of school cohort
University of Cairo	Egypt	1 590	15.95	1 366	85.91	19	1.19	40	2.52	165	10.38
Ain Shams University	Egypt	1 282	12.82	1,116	87.05	25	1.95	33	2.57	108	8.42
University of the Witwatersrand	South Africa	994	9.98	912	91.75	17	1.71	19	1.91	46	4.63
University of Alexandria	Egypt	916	9.17	789	86.14	33	3.60	24	2.62	70	7.64
University of Ibadan	Nigeria	738	7.37	633	85.77	13	1.76	16	2.17	76	10.30
University of Cape Town	South Africa	614	6.15	506	82.41	59	9.61	11	1.79	38	6.19
University of Nigeria	Nigeria	492	4.92	440	89.43	4	0.81	21	4.27	27	5.49
University of Lagos	Nigeria	475	4.74	408	85.89	8	1.68	21	4.42	38	8.00
University of Ghana	Ghana	434	4.33	425	97.93	0	0.00	0	0.00	9	2.07
Addis Ababa University	Ethiopia	243	2.43	232	95.47	4	1.65	1	0.41	6	2.47
University of Benin	Nigeria	235	2.35	211	89.79	4	1.70	14	5.96	6	2.55
University of Khartoum	Sudan	184	1.85	170	92.39	5	2.72	6	3.26	3	1.63
Obafemi Awolowo University	Nigeria	184	1.84	157	85.33	2	1.09	11	5.98	14	7.61
Assiut University	Egypt	140	1.40	134	95.71	2	1.43	2	1.43	2	1.43
Makerere University	Uganda	139	1.39	81	58.27	54	38.85	1	0.72	3	2.16
University of Pretoria	South Africa	122	1.22	116	95.08	0	0.00	2	1.64	4	3.28
University of Nairobi	Kenya	121	1.21	112	92.56	5	4.13	1	0.83	3	2.48
Kwame Nkrumah University of Science & Technology	Ghana	117	1.17	111	94.87	0	0.00	2	1.71	4	3.42
University of Ilorin	Nigeria	117	1.17	103	88.03	0	0.00	5	4.27	9	7.69

^a^Domestic means students who hold citizenship from the same country as their medical school is located in

This analysis led us to consider four distinct categorizations of African-trained physicians migrating to the US: (1) citizens from an African country who attended medical school in their own country; (2) citizens from an African country who attended medical school in another African country; (3) US citizens who attended medical school in an African country; (4) citizens from a country outside Africa, and other than the United States, who attended medical school in an African country. Based on 2015 data, most of African-education IMGs were in category 1 (86.2%, *n =* 11 697), followed by category 4 (7.5%, *n =* 1013), category 3 (4.0%, *n =* 537), and category 2 (2.3%, *n =* 317). This distribution was similar for 2005 data, although actual numbers varied. There are some differences between 2005 and 2015, most notably an increased number of schools with more than 100 graduates currently practicing in the US. This is a reflection of wider migration patterns for IMGs to the US. Also, some schools who appear in the 2015 list were not in operation in 2005.

## Discussion

The purpose of this study was to look, at two time points, at the demographics of African-trained physicians who migrated to the US.

We distinguish four different patterns of migration based on citizenship and medical school attended.^2^ As noted by other authors [5, 9], African-educated IMGs make up a relatively small proportion of the US workforce. The number of African-educated IMGs in the US has increased substantially in the past 10 years, as has the total number of practicing physicians. A proportion of these individuals were not citizens from African countries when they first enrolled in medical school. Moreover, the majority of African-educated IMGs attended medical school in a limited number of countries and, based on 2015 data, one third of them graduated from one of three (Northern African) medical schools. While these findings highlight some trends, more studies are needed to understand the “internationalization” of African medical schools and the potential role of geopolitical and economic events in shaping physician migration.

Our investigation provides unique information to characterize the so-called brain drain and the role of particular countries and institutions in supplying physicians for non-domestic markets. Effectively ignored in previous investigations, a substantial proportion of African-educated IMGs are citizens of countries other than where they trained, with many being citizens from non-African countries. While this does not diminish the potential healthcare impact of physician migration, it helps to explain the changing dynamics of medical education on the African continent. Similar to other places in the world, students may choose their educational programs not based on the local need for practitioners, but based on admission policies and personal economic considerations [21]. To the extent that medical education is publicly funded, the migration of these individuals represents both a financial and workforce loss for these countries.

The potentially negative impact of this educational model on both the local workforce and the provision of medical care in the country of education raises some important ethical questions. When medical schools in low-income countries educate students from other countries, only to have them leave the country after graduation, they may, in effect, be limiting their ability to address local physician supply issues. Even though foreign medical students may pay more for their education, effectively subsidizing local educational efforts, the capacity to educate a sufficient number of doctors, which is dependent on the number of matriculating students, is a limiting factor in developing an adequate local workforce. In Africa, where the number of medical schools is limited and the size of graduating classes may also be small (e.g., Uganda, Malawi) [20], the education of foreign nationals, while potentially economically advantageous, and not necessarily contributing to “brain drain”, can constrain the local workforce and have negative repercussions for the local healthcare systems. As noted by the WHO, there is no health without an adequate local workforce [22].

Our study enumerates the migration patterns of African-educated physicians to the US; it is important to look at this in relation to factors that shape migration in general, and “brain drain” of health professionals in particular. These are typically framed as a combination of push and pull factors [23, 24], and include macroeconomic dynamics such as market segmentation and labour market configurations, political environment such as good governance and security from armed conflict, personal economic calculations such as wages, and professional considerations such as advanced training options and working conditions. Simply put, the literature suggests that migration is spurred by the prospects of advanced training, more attractive salaries and working conditions, and a higher standard of living [25–27]. In light of this, it is not surprising that there has been an increase in the number of African-educated physicians migrating to the US. The quality of graduate medical training in the US is perceived to be among the best in world and conflicts in some nations (e.g., South Sudan, Libya) would certainly incentivize some graduates to leave.

Between 2005 and 2015, there has been an increase in the number of African-educated physicians coming to the US for graduate medical education and practice opportunities. However, depending on a number of supply and demand issues, future opportunities may be limited. In the US, the Association of American Medical Colleges (AAMC) has estimated that there will be a shortage of 91 500 doctors by 2020 and 130 600 by 2025 [28]. To overcome this deficit, new US medical schools have opened and are under development [29]. However, with no substantial increase in the number of graduate training positions, which seems likely given the required funding [30], the number of graduates from US medical schools may eventually surpass the number of residency positions. Because US medical graduates tend to remain in their home country for specialty training, the number of IMGs who train in the US is expected to decrease [31–34]. While the impact of these policy changes on the migration of African-educated IMGs to the US remains to be seen, the principal “pull factor” of advanced training opportunities may not be as relevant in the future.

Unlike previous investigations, we were able to determine the citizenship of US physician immigrants who attended medical school in an African country. Not only did a few medical schools account for a substantial number of migrants, but many of the graduates from these schools held citizenship from another country, most from countries outside of Africa. While these IMGs are African-educated, it is uncertain whether they enrolled in medical school with the intention to practice locally. A similar situation exists in Central America and the Caribbean where medical schools take a disproportionate number of non-domestic applicants considering the local labor market [35, 36]. For some schools, such as the St Christopher Iba Mar Diop College of Medicine, where 98% of graduates who currently practice in the US were non-domestic non-African citizens, it seems highly unlikely that the educational system is being driven by local healthcare needs. Even for countries that do supply a considerable number of graduates to the US physician workforce, such as Egypt, the large number of medical schools located there, and sizeable enrolment [37], may be indicative of the development of business models that encourage the export of physicians, as has been done in other regions of the world [38]. Our data show that 40.0% of African-educated physicians working in the US graduated from North African medical schools and that in total 11.4% of African-trained IMGs held citizenship of countries outside Africa. These graduates are not really contributing to the “brain drain” that impacts healthcare in SSA, except for the fact that they are taking medical school positions that could be better utilized for strengthening the local workforce. Clarifying the role of these African-trained physicians, in consideration to the “brain drain” debate, is essential if local, national, and international policies are to be put in place to address the global maldistribution of physicians and other healthcare workers.

There are a number of limitations of our investigation. First, while we looked specifically at African emigration to the US, it is important, from a total “brain drain” perspective, to note that African graduates also migrate elsewhere [39, 40]. The full extent of African physician emigration requires data from all receiving nations. Second, there are known issues with the AMA-Master file including under/overcounting physicians in different practice settings or specialties [41]. Despite this, the AMA has been the best available source for US physician workforce data [9, 42–44]. Third, for the comparisons, we looked separately at the 2005 and 2015 cohorts. While it is difficult, yet possible, to link individual physicians at the two time points, enabling the identification of new émigrés and, potentially, those who left practice, we were not interested in following individual practitioners over time. This longitudinal tracking of immigrant physicians would be helpful in terms of quantifying reverse migration, especially among those physicians who come to the US solely for Graduate Medical Education; however, the AMA Masterfile we used does not contain such data. Based on previous investigations, however, this reverse migration, at least from the US back to the country of origin (country of medical school education), is likely to be quite low [33, 45]. Further research could address African-educated physicians’ tenure in the US medical system.

Additional limitations include lack of information on the quality of the medical schools, including curricula and competencies addressed, and the lack of comparative USMLE performance data for the African-educated physicians in our study. We were not able to establish whether the standards of education in the dominant source countries changed between 2005 and 2015, which might provide additional insight into reasons for migration. We did not determine the USMLE performance by medical school as the number of African-educated physicians taking USMLE is small for some schools and we have no method to establish the denominators, e.g., how the students leaving for the US compare to the rest of their cohort.

Putting aside the ethics of international migration of physicians [46–50], it is important to know who is leaving, where they are coming from, and where they are going. This information can be used by governments of African nations and supranational organizations such as the World Health Organization to better support African health workforce capacity efforts. While we were able to quantify the emigration of African-educated physicians to the US, future research should focus on why certain countries, and schools within these countries, are the primary source for these physicians. On a school level, research should concentrate on medical education funding models, including incentives to enroll non-domestic students and retention schemes for domestic students. While some individuals who emigrate may eventually return to their home country, and others may make reciprocal financial contributions in the future, the exodus of well-trained medical graduates is certain to challenge the educational and healthcare systems in some countries, especially those with disproportionally large burdens of disease [51]. While efforts aimed at strengthening the health systems can be productive, promoting health workforce retention will be difficult if many of those enrolled in the educational programs never had the intention of practicing locally.

## Conclusions

The actual outflow of African-educated physicians to the US has increased over the past 10 years but their contribution to “brain drain” appears uneven across regions or countries.

The majority of African graduates migrating to the US come from relatively few countries, and from a limited number of medical schools. A substantial proportion were not citizens of African countries when they attended medical school. These findings highlight the need for more specific studies or local and national labour dynamics, including the economics of medical education. When combined with analyses from other countries (e.g., Australia, Canada, United Kingdom), they can help inform national and international workforce policies.

## Footnotes

1 Countries not included in sub-Saharan Africa but on the African continent are Algeria, Egypt, Libya, Morocco, Tunisia, and Western Sahara.

2 A fifth pattern exists, but is not verifiable with our data. Citizens from an African country who attended medical school in a country that is not their own, and is not on the African continent, and currently practice in the US.
