# Correction to: a comparison of physician emigration from Africa to the United States of America between 2005 and 2015

**DOI:** 10.1186/s12960-017-0251-y

**Published:** 2017-10-24

**Authors:** Robbert J. Duvivier, Vanessa C. Burch, John R. Boulet

**Affiliations:** 1 Foundation for Advancement of International Medical Education and Research (FAIMER), Philadelphia, United States of America; 2 Medical Education Unit, School of Medicine and Public Health, University of Newcastle, University Drive, Callaghan, NSW 2308 Australia; 3 Department of Medicine, Faculty of Health Sciences, University of Cape Town, Cape Town, South Africa

## Correction

Table 2 in the published version of this article [1] is incorrect and contains some typographical errors. The table is shown as it should be below.

**Table 2:** African-educated IMGs in the US physician workforce by country of citizenship at entry to medical school

	2005		2015		
	Number	% of total	Number	% of total	% change
EGYPT	3655	34.27	4189	30.92	+14.61
**NIGERIA** ^a^	2203	20.66	3232	23.85	+46.71
**SOUTH AFRICA**	1633	15.31	1485	10.96	−9.06
**GHANA**	547	5.13	730	5.39	+33.46
**ETHIOPIA**	355	3.33	666	4.92	+87.61
UNITED STATES OF AMERICA	313	2.94	537	3.96	+71.57
JORDAN	220	2.06	201	1.48	−8.64
**SUDAN**	218	2.04	410	3.03	+88.07
UNITED KINGDOM	180	1.69	208	1.54	+15.56
**KENYA**	151	1.42	206	1.52	+36.42
INDIA	140	1.31	184	1.36	+31.43
**ZIMBABWE**	113	1.06	122	0.9	+7.96
**UGANDA**	90	0.84	114	0.84	+26.67
LIBYA	78	0.73	278	2.05	+256.41
ALGERIA	77	0.72	100	0.74	+29.87
PAKISTAN	60	0.56	71	0.52	+18.33
MOROCCO	59	0.55	96	0.71	+62.71
**CAMEROON**	52	0.49	105	0.77	+101.92
SYRIA	52	0.49	72	0.53	+38.46
LEBANON	48	0.45	45	0.33	−6.25
Other sub-Saharan	173	1.62	200	1.48	+15.61
Other African (non sub-Saharan)	59	0.55	64	0.47	+8.47
Other (non-African)	188	1.76	234	1.73	+24.47
Total sub-Saharan^a^	5535	51.90	7270	53.66	+31.35
Total African (non sub-Saharan)	3928	36.83	4727	34.88	+20.34
Total other (non-African)	1201	11.26	1552	11.45	+29.23

^a^nations in **bold** are considered Sub-Saharan Africa total numbers for Table 2 differ from Table 1 because of missing citizenship information <0.05% of the physicians did not have citizenship information coded in the ECFMG database
